# Anatomical study and clinical significance of the posterior ramus of the spinal nerve of the lumbar spine

**DOI:** 10.3389/fcell.2022.1019309

**Published:** 2022-10-03

**Authors:** Zhenfeng Zhang, Jing Liu, Yejie Xu, Zeyan Chen, Shiwen Luo, Xin Zhang, Guoliang Wang, Liang Cheng

**Affiliations:** ^1^ Department of Orthopedics, Guangzhou Development District Hospital, Guangzhou, China; ^2^ Department of Orthopedics, The Third Affiliated Hospital of Southern Medical University, Guangzhou, China; ^3^ Department of Anatomy, School of the Basic Medical Sciences, Southern Medical University, Guangzhou, China; ^4^ Department of Orthopaedics and Traumatology, Wuyi Hospital of Traditional Chinese Medicine, Jiangmen, China

**Keywords:** low back pain, posterior ramus of the spinal erve, anatomy, nerve block, ultrasounds, transverse

## Abstract

**Background and objectives:** Chronic nonspecific back pain is a common clinical disease typically treated by ultrasound-guided spinal injection. This minimally invasive treatment targets the posterior ramus of the spinal nerve (PRSN). The target of the medial branch is clear, but there is unclear target for the intermediate and lateral branches. This study attempted to observe the distribution of PRSN in the dorsal region of transverse process to provide a more detailed anatomical basis for treating spinal pain.

**Methods:** The present study was conducted on 16 transverse processes of six adult male embalmed corpses. The dorsal area of the transverse process was divided into three equal zones, which are zone I, zone II and zone III from inside to outside. The origin, distribution, quantity, transverse diameter, and relationship with the bone structure of the PRSN on the transverse process were observed.

**Results:** Sixty PRSNs were found in the lumbar of six cadavers, of which 48 were divided into three branches, and 12 PRSNs were divided into two branches. The intermediate branch is mainly distributed in zone I, and the lateral branch is mainly distributed in zone II. Twenty-nine communicating branches were found in 48 adjacent segments of six specimens, all of which originated from the intermediate branch of the previous segment and connected with the lateral branch of the next segment.

**Conclusion:** This anatomical study describing the PRSN may have important clinical significance for spinal surgeons. Understanding the bony localization targets of the PRSN and the links between the PRSNs may benefit patients with low back pain who receive spinal injections.

## Highlights

The target of spinal pain injection therapy is the posterior ramus of the spinal nerve, previous anatomical studies have confirmed the injection target of medial branch, but the injection targets of intermediate branch and lateral branch are still unclear. We found that in the dorsal region of transverse process, the intermediate branch is mainly distributed in zone I and the lateral branch is mainly distributed in zone II. A communicating branch exists in adjacent segment. This study analyzed the posterior ramus of the spinal nerve distribution on the transverse process to provide a more detailed anatomical basis for the treatment of spinal pain.

## Introduction

Nonspecific chronic lower back pain is the leading cause of disability worldwide (Hoy et al., 2014), with at least 80% of the population experiencing lower back pain in their lifetime (Rubin, 2007) and approximately 5%–10% of those progressing to chronic symptoms (Meucci et al., 2015). The condition may affect the quality of life and cause psychological problems such as pain-related disability, poor sleep, depression, and anxiety (Gatchel et al., 2008). However, the increasing expenditure on spinal injection therapy and medical treatment has aggravated the global economic burden (JannaChan and Richard, 2007). The posterior ramus of the spinal nerve (PRSN) has received increasing attention for diagnosing and treating lower back pain and preventing disabling sequelae after spinal surgery. Zhou et al. (2012) proposed that the PRSN was the cause of nonspecific chronic lower back pain. The affected PRSN could be located and treated according to anatomical and clinical manifestations (Zhou et al., 2012; Kim et al., 2014). The syndrome is usually diagnosed with a nerve block and treated with minimally invasive surgery (Manchikanti et al., 2010; Manchikanti et al., 2013; Kim et al., 2014; McCormick Zachary et al., 2015; Kreiner et al., 2020). However, the positive rate of PRSN has been revealed to vary across studies, and the long-term efficacy of injection, acupuncture, and laser acupuncture is poor (Kreiner et al., 2020).

The PRSN is usually small and distributed in the deep muscles and corresponding parts of the skin of the nape, back, waist, and buttocks, and its primary branching pattern remains controversial (Bogduk et al., 1982; KatsuraSato, 2002; Zhu et al., 2021). Ultrasound-guided treatment is recommended for a better therapeutic outcome of the PRSN syndrome (Wang, 2018); however, accurate positioning is the key to treatment. Saito et al. (2013) confirmed that the medial branch (MB) lies consistently between the accessory and the mammillary processes in the lumbar region and made a model diagram of PRSN, but the precise bony positioning of the intermediate branch (IB) and lateral branch (LB) remains unclear.

Therefore, the present study attempted to further anatomize and observe the distribution of PRSN in the dorsal region of transverse process and discuss its possible clinical significance to provide a more detailed anatomical basis for treating spinal pain.

## Materials and methods

The present study involved six adult male embalmed cadavers (age range: 57–83 years; mean age: 69 years) without local disease, surgery, or injury to the lumbar region and was approved by the Ethics Committee of Guangzhou Development District Hospital (GZ2022006. 27 April 2022). All specimens were collected and processed by the Teaching and Research Section of the Department of Anatomy, Southern Medical University, according to relevant scientific research, laws, and other regulations.

The origin, distribution, quantity, and transverse diameter of the PRSN of the L1–L5 transverse process and its relationship with bone structure (60 transverse processes in total) were studied.

**FIGURE 1:**
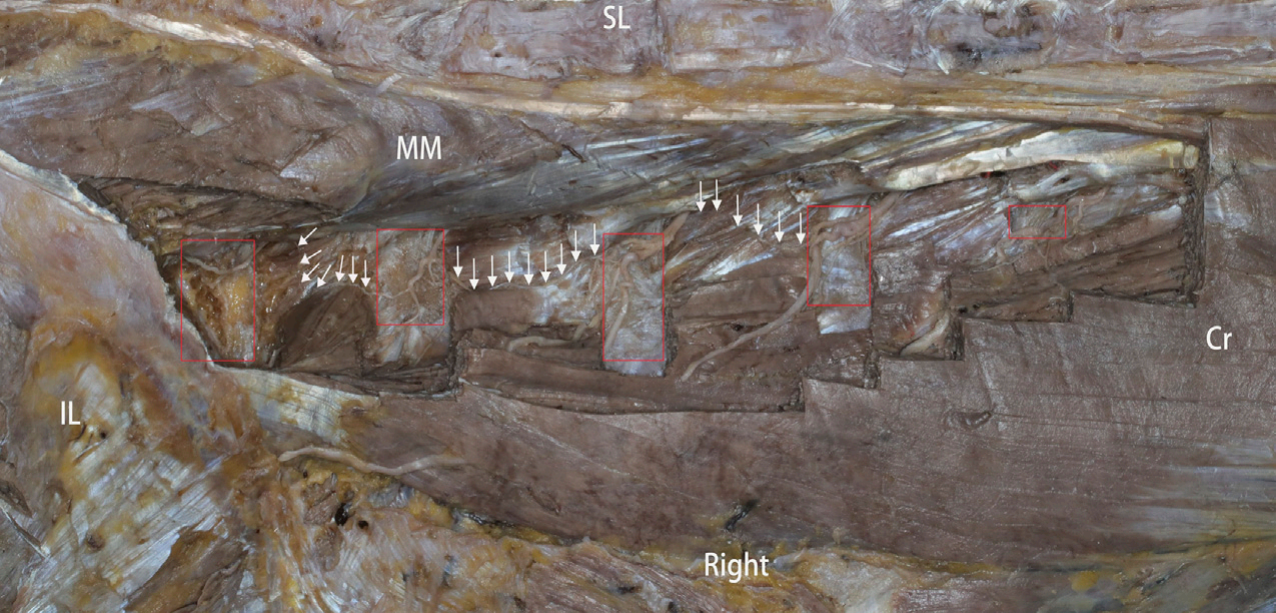
Photo showing a dor`sal side view. SL, supraspinous ligament; MM, multifidus muscle; Cr, cranial; IL, ilium. The red rectangle represents the transverse process; the arrow (“→”) denotes the intersegment traffic branch.

**FIGURE 2:**
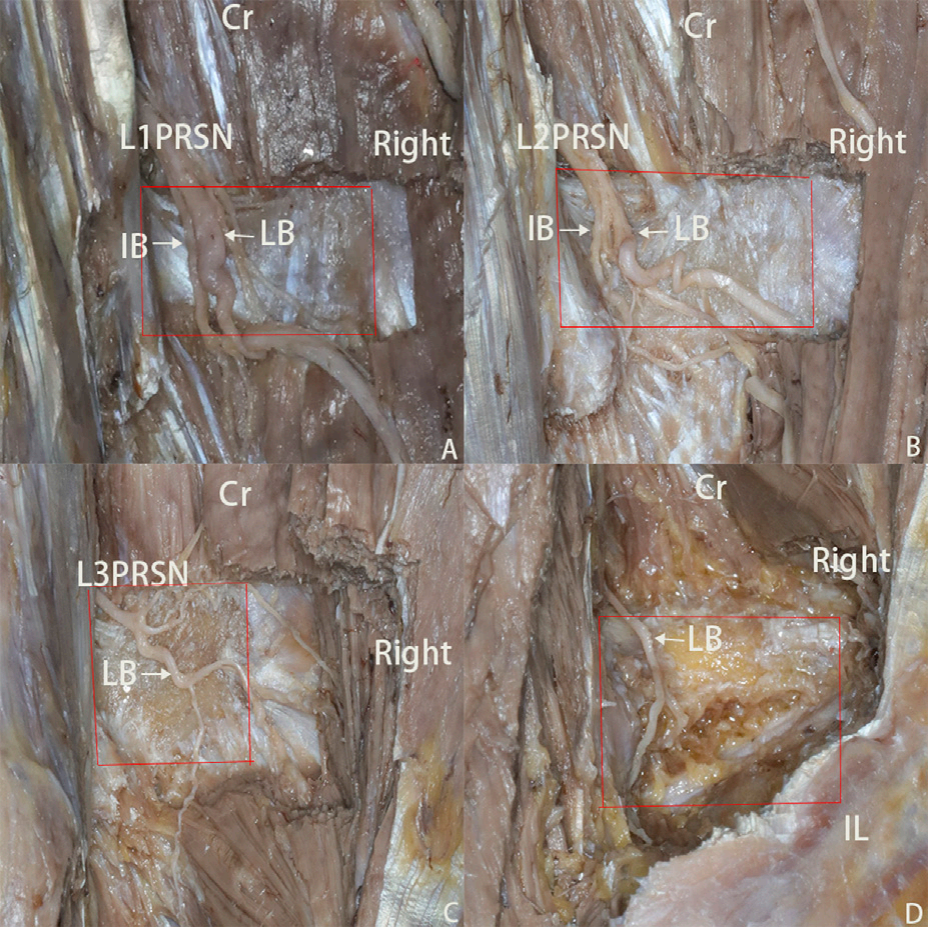
**(A–D)** photos displaying dorsal side of L2-L5 transverse process. Cr, cranial; IB, intermediate branch; LB, lateral branch. The red rectangle represents the transverse process; the yellow arrow denotes the traffic branch.

The corpses were placed in a prone position, the lower back skin was incised, and the superficial fascia, fat, latissimus dorsi, and serratus posterior inferior were dissected to expose the erector spinae. In the lumbar intervertebral foramen area of L1–L5, the muscles were removed layer by layer by slotting, and the PRSN was exposed (Figure 1). The dissection was performed under a surgical microscope (Carl Zeiss, Jena, Germany) to identify and preserve all nerve branches and adequately expose them and the transverse process (Figure 2).

**FIGURE 3:**
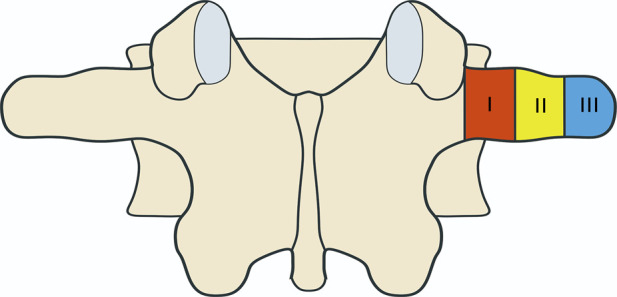
A diagram showing the division of the dorsal area of transverse process. The dorsal area of transverse process is divided into three zones. The zone near the posterior midline was defined as zone I, the middle zone as zone II, and the area near the outside as zone III.

The dorsal area of the transverse process was divided into three equal zones. The zone near the posterior midline was defined as zone I, the middle zone as zone II, and the area near the outside as zone III (Figure 3). The number, origin, and spatial orientation of nerves were checked in each area of the L1–L5 transverse process, photos were taken with a camera, and the diameter of the nerves was measured with vernier calipers accurate to 0.1 mm (Nscing Es, Nanjing, China).

The measurements were analyzed using SPSS software version 26.0 (IBM SPSS Statistics for Windows; IBM) and expressed in the form of “ $\bar{x}$ ± s (minimum/maximum)”.

## Results

### Types of primary branches of the posterior rami of spinal nerves

Sixty PRSNs were found in the lumbar intervertebral foramina of six cadavers, and 48 (80%) PRSNs were divided into three main branches, namely MB, IB, and LB. Twelve (20%) PRSNs were divided into two main branches, MB and LB. In L2 and L4 segments, 12 (100%) PRSNs were divided into three main branches; in L1 and L3 segments, ten (83.3%) PRSNs were divided into three branches, and two (16.7%) PRSNs were divided into two branches. In the L5 segment, four (33.3%) PRSNs were divided into three branches, and eight (66.7%) PRSNs were divided into two branches. The types and numbers of the posterior rami of the spinal nerve are displayed in Table 1.

**TABLE 1:** The branching pattern of the posterior ramus of the spinal nerve.

Segment	Three branches	Two branches
L1	10	2
L2	12	0
L3	10	2
L4	12	0
L5	4	8

Sixty PRSNs of all segments were investigated (unit: PRSNs).

### Distribution of the intermediate branch on the dorsal side of the transverse process

Forty-eight intermediate branches passed through the back of 16 transverse processes in six specimens, of which ten IB passed through the back of the L1 transverse process, four (40%) in zone I and six (60%) in zone II). Twelve IB passed through the back of the L2 transverse process, nine (75%) in zone I and three (25%) in zone II. Ten IB passed through the back of the L3 transverse process, all in zone I. Twelve IB passed through the back of the L4 transverse process, ten branches (83.3%) in zone I and two branches (16.7%) in zone Ⅱ. Four IB passed through the back of the L5 transverse process, all in area I. The diameter, number, and distribution of the IBs are displayed in Table 2.

**TABLE 2:** The measurement of the intermediate branch and lateral branch in the lumbar region “x ± s (minimum–maximum).”

Intermediate branch				Lateral branch			
Segment	Part	Number	Diameter (mm)	Segment	Part	Number	Diameter (mm)
L1	I	4	0.5 ± 0.4 (0.2–1.1)	L1	I	1	1
	II	6	1.3 ± 0.5 (0.4–1.7)		II	7	0.6 ± 0.2 (0.3–0.8)
	III	—	—		III	4	0.4 ± 0.2 (0.2–0.6)
L2	I	9	0.5 ± 0.3 (0.3–1.2)	L2	I	—	—
	II	3	0.4 ± 0.5 (0.1–1)		II	11	1.4 ± 0.5 (0.7–2.3)
	III	—	—		III	1	0.2
L3	I	10	0.6 ± 0.3 (0.2–0.9)	L3	I	2	1.0 ± 0.2 (0.8–1.1)
	II	—	—		II	10	0.8 ± 0.4 (0.2–1.5)
	III	—	—		III	—	—
L4	I	10	0.3 ± 0.2 (0.1–0.7)	L4	I	2	0.3 ± 0.1 (0.2–0.3)
	II	2	0.4 ± 0.4 (0.1–0.7)		II	8	0.3 ± 0.2 (0.1–0.6)
	III	—	—		III	2	0.3 ± 0.1 (0.2–0.3)
L5	I	4	0.2 ± 0.1 (0.1–0.3)	L5	I	4	0.2 ± 0.1 (0.1–0.2)
	II	—	—		II	7	0.2 ± 0.1 (0.1–0.3)
	III	—	—		III	1	0.3

### Distribution of lateral branch on the dorsal side of the transverse process

Sixty lateral branches passed through the back of 16 transverse processes in six specimens, of which 12 LB passed through the back of the L1 transverse process, one (8.3%) in zone I, seven (58.3%) in zone II, and four in zone III (33.3%). Twelve LB passed through the back of the L2 transverse process, 11 (91.7%) in zone II and 1 (8.3%) in zone III, and 12 LB passed through the back of the L3 transverse process, two (16.7%) in zone I and ten (83.3%) in zone II. Twelve LB passed through the back of the L4 transverse process, two (16.7%) in zone I, eight (66.7%) in zone II, and two (16.7%) in zone III. Twelve LB passed through the back of the L5 transverse process, four (33.3%) in zone I, seven (58.3%) in zone II, and one (8.3%) in zone III. The diameter, number, and distribution of the LB are illustrated in Table 2.

### Traffic in the posterior rami of the spinal nerve

Twenty-nine communicating branches were found in 48 adjacent segments of six specimens, all of which originated from the middle branch of the previous segment and connected with the posterolateral branch in the next segment (see Figure 1). The L1-2 segment has six traffic branches, the L2-3 segment has ten traffic branches, the L3-4 segment has nine traffic branches, and the L4-5 segment has four traffic branches.

## Discussion

We found that in the dorsal region of transverse process, the intermediate branch is mainly distributed in zone I and the lateral branch is mainly distributed in zone II. A communicating branch exists in adjacent segment. This study analyzed the posterior ramus of the spinal nerve distribution on the dorsal side of transverse process to provide a more detailed anatomical basis for the treatment of spinal pain.

### The branching pattern of the posterior ramus of the spinal nerve

The primary branching pattern of the lumbar PRSN is still controversial. Previous textbooks and studies have divided the PRSN into two main branches, the medial and the lateral (Bogduk et al., 1982; Cohen and Raja, 2007; Berry et al., 1995; ten Donkelaar et al., 2018). Bogduk et al. (1982) was the first anatomist to accurately describe the PRSN and divided the PRSN into three branches: MB, IB, and LB. Saito et al. (2019) conducted an anatomical study using the ventral approach and found that most of the PRSN in the lumbar segment was divided into three branches, of which the L3 and L4 segments were all divided into three branches, and the L5 segment also had three branches. Similarly, our careful dissection of cadavers through the dorsal approach revealed that the PRSN is mainly divided into three primary branches in the L1–L4 segment and two primary branches in the L5 segment; therefore, there is no fixed pattern for the primary branches of PRSN.

### Clinical relevance of posterior ramus of the spinal nerve

Spinal dorsal ramus mediated back pain is the second most commonly described condition originating from pathology involving posterior branches of lumbar spinal nerves (Kozera et al., 2017). Bogduk (1980) proposed the concept of the lumbar dorsal ramus syndrome in 1980. The dorsal rami of the spinal nerve contain sensory fibers from the skin, subcutaneous and deep tissues, and viscera (Sharma et al., 2021). The MB innervates the area from the midline to the facet joint line, the IB supplies the longissimus muscle, and the LB supplies tissue outside the facet joints (Shao, 1992; Saito et al., 2006; Kozera and Ciszek, 2016). Selective dorsal branch block or amputation is equivalent to interrupting the path of sensory signals to the brain, thereby reducing pain. Therefore, the precise positioning of the PRSN is particularly important.

Previous studies have confirmed that the MB lies consistently between the accessory and the mammillary processes in the lumbar region. Kennedy et al. (2018) proposed that the junction of the superior articular process and the transverse process could be the target of MB injection therapy. The clinical use of ultrasound-guided technology for targeted spinal injection has increased with bedside portable ultrasound, as this technology can reduce the medical burden of radiation guidance and avoid radiation exposure. At present, MB injection under the guidance of B-ultrasound has been widely used in clinical practice, but the therapeutic targets of the IB and the LB are still unclear, and there is a lack of relevant clinical data. Anthony and Satishchandra (2014) demonstrated considerable variability in the location of the MB and LB of the dorsal branch through a cadaveric study. The present study revealed that on the dorsal side of the transverse process, the IB is mainly distributed in zone I, and the LB is mainly distributed in zone II. On the one hand, if the surgeon separates the erector spinae too far or operates in the transverse process area, the PRSN may be damaged, resulting in sensory or motor dysfunction. Therefore, when the surgical site involves the vicinity of transverse process, the PRSN should be carefully identified. On the other hand, we can identify the dorsal side of transverse process by ultrasound and X-ray machine and divide it into three equal zones. According to the anatomical results, we can make a diagnostic block test on the primary branch of the PRSN. If the diagnosis result is positive, we can consider accurately cutting the primary branch of the PRSN under percutaneous endoscope. Park and Kwak (2014) found that injection therapy at transverse process fractures can significantly relieve the patient’s low back pain, which confirms our conjecture. We hypothesized that the displacement of the fracture end stimulates the IB and LB to cause pain, and the injection therapy interrupts the pain signal conduction to achieve an analgesic effect.

**FIGURE 4:**
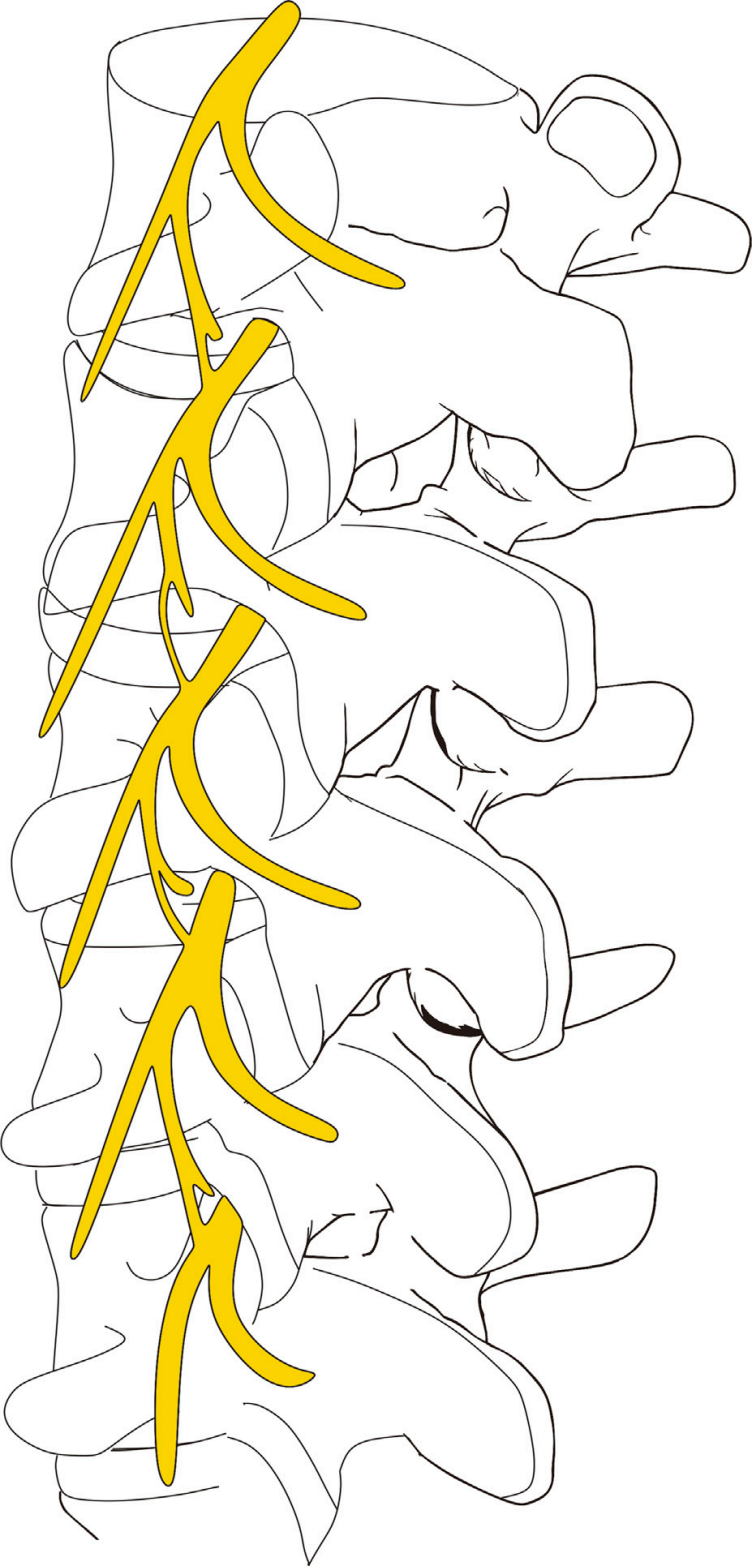
Examples of a digital model of the posterior rami of the lumbar spinal nerve.

There are reports that rhizotomy failed to relieve the symptoms of low back pain (Sindou et al., 2001), which may be related to the existence of communicating branches between the posterior branches of adjacent spinal nerves. Steinke et al. (2009) found through anatomy that the intermedial branch develops a short ventral branch, whereas the dorsal branch is long. The short ventral branch of the intermedial branch of the PRSN innervates the an intermediate muscular compartment from the ventral side and the dorsal innervates the an intermediate muscular compartment from the dorsal side. We not only found that the intermedial branch was divided into two branches, but also found that the short ventral branch of the previous segment was connected to the LB of the next segment (Figure 4). If blocking the LB may not completely block the transmission of pain signals, we suggest blocking the IB and LB of the same segment simultaneously; however, this requires further clinical verification.

Our study is based on morphological observations; the measured data and texture of embalmed carcasses may differ from living tissues, the measurement of vernier caliper may be biased, and nerves may shift during dissection. Furthermore, the small sample size, the time taken for careful dissection of each part, and the sex inequality in the study (cadavers were all adult males) prevent the generalization of the findings to the entire population. However, despite these limitations, we believe the study findings complement the anatomical knowledge of the PRSN, thus benefiting clinicians and patients with pain associated with the PRSN.

## Conclusion

This systematic anatomical study describing the PRSN in the dorsal region of transverse process may have important clinical significance for spinal surgeons and pain doctors. Understanding the bony localization targets of IB and LB, as well as the connections between the PRSN, may bring significant benefits to patients with low back pain who receive a spinal injection.

## Data Availability

The original contributions presented in the study are included in the article/supplementary material, further inquiries can be directed to the corresponding authors.

## References

[B1] Anthony Y. Satishchandra G. Endoscopically guided foraminal and dorsal rhizotomy for chronic axial back pain based on cadaver and endoscopically visualized anatomic study [J」.Int. J. Spine Surg., 2014, 8: 23. 10.14444/1023 PMC4325504 25694936

[B2] Berry M. H. Standring S. M. Bannister L. H.: Dorsal rami of the spinal nerves, Gray’s Anatomy, 38th edition. Edited by Bannister L. H. Berry M. M. Collins P. Dyson M. Dussek J. F. Ferguson M. W. J..London, Churchill Livingstone, 1995, pp 1261–1263.

[B3] Bogduk N. Lumbar dorsal ramus syndrome. Med. J. Aust. 1980;2(10):537–541. 10.5694/j.1326-5377.1980.tb100759.x 6450875

[B4] Bogduk N. Wilson A. S. Tynan W. The human lumbar dorsal rami. J. Anat. 1982;134(2):383–397. 7076562 PMC1167925

[B5] Cohen S. P. Raja S. N. Pathogenesis, diagnosis, and treatment of lumbar zygapophysial (facet) joint pain. Anesthesiology 2007; 106: 591–614. 10.1097/00000542-200703000-00024 17325518

[B7] Gatchel R. J. Bernstein D. Stowell A. W. Pransky G. Psychosocial differences between high-risk acute vs. chronic low back pain patients. Pain Pract. 2008;8:91–97. 10.1111/j.1533-2500.2008.00176.x 18366464

[B9] Hoy D. March L. Brooks P. Blyth F. Woolf A. Bain C. The global burden of low back pain: Estimates from the Global Burden of Disease 2010 study. Ann. Rheum. Dis. 2014;73(6): 968–974. 10.1136/annrheumdis-2013-204428 24665116

[B6] Janna Friedly Chan Leighton Richard Deyo. Increases in lumbosacral injections in the medicare population: 1994 to 2001.[J]. Spine, 2007, 32(16): 10.1097/BRS.0b013e3180b9f96e 17632396

[B8] Katsura Higuchi Sato Tatsuo. Anatomical study of lumbar spine innervation. Folia Morphol., 2002, 61, 71, 79.(2): 12164053

[B10] Kennedy D. J. Huynh L. Wong J. Mattie R. Levin J. Smuck M. Corticosteroid injections into lumbar facet joints: A prospective, randomized, double-blind placebo-controlled trial. Am. J. Phys. Med. Rehabil. 2018;97(10):741–746. 10.1097/PHM.0000000000000960 29734232

[B11] Kim S. J. Ko M. J. Lee Y. S. Park S. W. Kim Y. B. Chung C. Unusual clinical presentations of cervical or lumbar dorsal ramus syndrome. Korean J. Spine 2014;11(2): 57–61. 10.14245/kjs.2014.11.2.57 25110484 PMC4124925

[B12] Kozera K. Ciszek B. Posterior branches of lumbar spinal nerves - Part I: Anatomy and functional importance. Ortop. Traumatol. Rehabil. 2016;18(1):1–10. 10.5604/15093492.1198827 27053304

[B13] Kozera K. Ciszek B. Szaro P. Posterior branches of lumbar spinal nerves-Part III: Spinal dorsal ramus mediated back pain-pathomechanism, symptomatology and diagnostic work-up. Ortop. Traumatol. Rehabil. 2017;19(4):315–321. 10.5604/01.3001.0010.4611 29086740

[B14] Kreiner D. S. Matz P. Bono C. M. Cho C. H. Easa J. E. Ghiselli G. Guideline summary review: An evidence-based clinical guideline for the diagnosis and treatment of low back pain. Spine J., 2020, 20(7):998–1024. 10.1016/j.spinee.2020.04.006 32333996

[B15] Manchikanti L. Abdi S. Atluri S. Benyamin R. M. Boswell M. V. Buenaventura R. M. An update of comprehensive evidence-based guidelines for interventional techniques in chronic spinal pain. Part II: Guidance and recommendations.Pain Physician 2013; 16: 49–283. 10.36076/ppj.2013/16/s49 23615883

[B16] Manchikanti L. Datta S. Derby R. Wolfer L. R. Benyamin R. M. Hirsch J. A. A critical review of the American Pain Society clinical practice guidelines for interventional techniques:Part 1. Diagnostic interventions. Pain Physician 2010; 13:141–174. 10.36076/ppj.2010/13/e141 20495596

[B17] McCormick Zachary L. Marshall Benjamin Walker Jeremy McCarthy R. Walega D. R. Long-term function, pain and medication use outcomes of radiofrequency ablation for lumbar facet syndrome. Int. J. Anesth. Anesth. 2015; 2(2): 028, 10.23937/2377-4630/2/2/1028 26005713 PMC4440581

[B18] Meucci R. D. Fassa A. G. Faria N. M. Prevalence of chronic low back pain: Systematic review. Rev. Saude Publica.2015;49, S0034, 10.1590/S0034-8910.2015049005874 PMC4603263 26487293

[B19] Park J. M. Kwak K. H. Clinical usefulness of fracture site *in situ* block on lumbar spine transverse process fracture. Pain Pract. 2014;14(8):752–756. 10.1111/papr.12213 24750583

[B20] Rubin D. I. Epidemiology and risk factors for spine pain. Neurol. Clin. 2007;25(2): 353–371. 10.1016/j.ncl.2007.01.004 17445733

[B21] Saito T. Steinke H. Hammer N. Li Z. L. Kawata S. Yasuda M. Third primary branch of the posterior ramus of the spinal nerve at the thoracolumbar region: A cadaveric study. Surg. Radiol. Anat. 2019;41(8):951–961. 10.1007/s00276-019-02258-z 31119410

[B22] Saito T. Steinke H. Miyaki T. Nawa S. Umemoto K. Miyakawa K. Analysis of the posterior ramus of the lumbar spinal nerve: The structure of the posterior ramus of the spinal nerve. Anesthesiology. 2013;118(1):88–94. 10.1097/ALN.0b013e318272f40a 23165471

[B23] Saito T. Yoshimoto M. Yamamoto Y. Miyaki T. Itoh M. Shimizu S. The medial branch of the lateral branch of the posterior ramus of the spinal nerve. Surg. Radiol. Anat. 2006;28(3):228–234. 10.1007/s00276-006-0090-3 16612554

[B24] Shao Z. H. Posterior spinal rami in localization of low back pain. Zhonghua Wai Ke Za Zhi. 1992;30(4):205–206.254.Chinese. 1473397

[B25] Sharma P. Kulkarni M. Gandotra A. Intra-dural intercommunications between dorsal roots of adjacent spinal nerves and their clinical significance. Surg. Radiol. Anat. 2021;43(9):1519–1526. 10.1007/s00276-021-02761-2 33961081

[B26] Sindou M. Mertens P. Wael M. Microsurgical DREZotomy for pain due to spinal cord and/or cauda equina injuries: Long-term results in a series of 44 patients. Pain. 2001;92(1-2):159–171. 10.1016/s0304-3959(00)00487-5 11323137

[B27] Steinke H. Saito T. Miyaki T. Oi Y. Itoh M. Spanel-Borowski K. Anatomy of the human thoracolumbar Rami dorsales nervi spinalis. Ann. Anat. 2009;191(4):408–416. 10.1016/j.aanat.2009.04.002 19570665

[B28] ten Donkelaar H. J. Kachlik D. Tubbs R. S. (2018) An illustrated terminologia neuroanatomica. Springer, Berlin 10.1002/ca.22809 27910135

[B29] Wang Dajie. Image guidance technologies for interventional pain procedures: Ultrasound, fluoroscopy, and CT. Curr. Pain Headache Rep., 2018, 22, 6, 10.1007/s11916-018-0660-1 1): 29374352

[B30] Zhou L. Schneck C. D. Shao Z. The anatomy of dorsal ramus nerves and its implications in lower back pain. Neurosci. Med. 2012; 3: 192–201. 10.4236/nm.2012.32025

[B31] Zhu W. Zhao Q. Ma R. Liu Z. Zhao J. Liu Z. Anatomical study of the innervation of different parts of the posterior ligamentous region of the sacroiliac joint. Reg. Anesth. Pain Med., 2021, 46, 410, 415. 10.1136/rapm-2020-102366 5): 33619182

